# Asymmetric dimethylarginine (ADMA) modulates frequency-dependent muscarinic antagonism of β-adrenergic regulation of rat papillary muscle contractility

**DOI:** 10.21203/rs.3.rs-10670314/v1

**Published:** 2026-08-13

**Authors:** Aleksei Averin, Alexey Glukhov, Miroslav Nenov

**Affiliations:** University of Wisconsin–Madison; University of Wisconsin–Madison; Temple University

**Keywords:** Asymmetric dimethylarginine, papillary muscle, contractility, isoproterenol, acetylcholine

## Abstract

Accumulating evidence implicates the endogenous nitric oxide synthase inhibitor asymmetric dimethylarginine (ADMA) in cardiovascular impairments. Although the detrimental cardiovascular effects of ADMA have largely been attributed to endothelial dysfunction, recent studies suggest that ADMA also directly affects cardiomyocyte function. However, the action of ADMA on myocardial contractility remains poorly understood. Here, we tested the hypothesis that continuous exposure to 10 μM exogenous ADMA alters the contractile responsiveness of rat right ventricular papillary muscles to sympathetic and parasympathetic stimulation using the β-adrenergic agonist isoproterenol (ISO, 1 μM) and the muscarinic agonist acetylcholine (ACh, 10 μM). We assessed force development, force-frequency relationship (FFR), contraction and relaxation kinetics, and post-rest potentiation (PRP). ADMA did not alter basal contractility or the force-frequency relationship but reduced force generation at high stimulation frequency. However, ADMA altered autonomic regulation of papillary muscle contraction in a frequency-dependent manner. Specifically, ADMA altered the inhibitory effect of ACh on ISO-induced positive inotropic and negative lusitropic responses, as well as on the ISO-mediated increases in the maximal velocities of contraction and relaxation. These effects were frequency dependent: ADMA attenuated the antagonistic effect of ACh on ISO responses at low stimulation frequencies but enhanced it at higher frequencies. Finally, ADMA attenuated ISO-induced potentiation of PRP while enhancing the inhibitory effect of ACh on PRP. Our findings identify a previously unrecognized mechanism by which ADMA directly modulates myocardial function through frequency- and time-dependent regulation of the interaction between β-adrenergic and muscarinic signaling, suggesting that elevated ADMA levels may contribute to abnormal ventricular contractile regulation in cardiovascular disease.

## Introduction

Asymmetric dimethylarginine (ADMA) is a well-established cardiovascular risk factor and an endogenous inhibitor of nitric oxide synthase (NOS) (Landim et al. 2009; Sibal et al. 2010). ADMA is generated during the proteolysis of proteins containing methylated arginine residues, which are formed through the action of protein-arginine methyltransferases (Sibal et al. 2010; Cooke and Ghebremariam 2011). Once released, free ADMA can either be excreted in the urine or metabolized by either dimethylarginine dimethylaminohydrolase type 1 (DDAH1) to citrulline and dimethylamine (Sibal et al. 2010; Cooke and Ghebremariam 2011) or by alanine-glyoxylate aminotransferase-2 to α-keto-Δ asymmetric dimethyl–guanidino valeric acid (Caplin et al. 2012; Rodionov et al. 2014). Elevated ADMA concentrations can significantly reduce nitric oxide (NO) production, resulting in endothelial dysfunction (Böger 2003). Accordingly, elevated plasma levels of ADMA are associated with a variety of cardiovascular diseases, including coronary artery disease, hypertension, and diabetes mellitus (Sydow and Münzel 2003). Moreover, ADMA has been identified as an independent predictor of cardiovascular mortality (Böger 2005; Böger et al. 2009; Schlesinger et al. 2016). Increased ADMA levels have also been reported in patients with heart failure, left ventricular hypertrophy, and atrial fibrillation (Dückelmann et al. 2007; Stamboul et al. 2015; Nemeth et al. 2015). These observations highlight the clinical importance of ADMA elevation and suggest that dysregulation of ADMA metabolism may contribute to both the development and progression of cardiovascular disease.

Although the detrimental cardiovascular effects of ADMA are commonly attributed to endothelial dysfunction and impaired NO bioavailability, growing evidence suggests that ADMA can also exert direct effects on cardiomyocytes. Pathology-driven changes in cardiomyocyte ADMA and DDAH1 activity appear to play an important role in cardiac remodeling and myocardial dysfunction. For example, cardiomyocyte-specific overexpression of DDAH1 attenuates angiotensin II-induced left ventricular remodeling and hypertrophy (Kopaliani et al. 2021). Similarly, treatment with recombinant DDAH1 improves post-ischemic cardiac function by restoring cardiomyocyte energetic and functional activity (Lee et al. 2022). Conversely, cardiomyocyte-specific deletion of DDAH1 promotes hypertrophic stress-induced cardiac hypertrophy and ventricular remodeling (Xu et al. 2017). In addition, a negative correlation between myocardial mechano-energetics efficiency and ADMA concentration has been reported (Cefalo et al. 2024). Together, these findings suggest that increased ADMA levels in cardiomyocytes may directly compromise myocardial performance. Consistent with this hypothesis, exogenous ADMA has been shown to impair myocardial contractility by decreasing papillary muscle force generation in both dose- and time-dependent manner (Xiong et al. 2021). However, despite increasing recognition of the direct myocardial effects of ADMA, important aspects of ADMA-mediated regulation of cardiac function remain poorly understood. In particular, it is not known whether ADMA can influence the autonomic regulation of cardiac contractility, which is primarily governed by the interplay between β-adrenergic and muscarinic signaling pathways. β-adrenergic stimulation enhances myocardial contractility and relaxation through activation of cAMP-dependent signaling pathways, whereas muscarinic stimulation typically exerts an opposing influence, mainly through inhibition of β-adrenergic signaling. This cholinergic–adrenergic interaction plays a critical role in the dynamic regulation of cardiac performance during physiological stress and disease. Notably, both β-adrenergic and muscarinic signaling pathways are functionally linked to NO production and NOS activity in the heart (Balligand 1999; Böger et al. 2000; Hare 2003; Massion et al. 2003; Martin et al. 2006). Therefore, elevated ADMA levels may alter autonomic regulation of myocardial function by modulating NOdependent signaling mechanisms within cardiomyocytes.

In the present study, we investigated the effects of direct ADMA exposure on myocardial contractile regulation using rat right ventricular papillary muscle (PM) preparations. Specifically, we assessed the isometric force of contraction, the force-frequency relationship, contraction kinetics, and post-rest potentiation of PM contractility during electrical stimulation alone (baseline) or in the presence of β-adrenergic stimulation with isoproterenol (ISO) and muscarinic stimulation with acetylcholine (ACh). Our results demonstrate that continuous exposure to ADMA does not significantly affect basal myocardial contractility or the force-frequency relationship but induces a negative inotropic effect at high stimulation frequency. We further show that ADMA markedly modulates cholinergic-adrenergic regulation of ventricular contraction. In particular, ADMA alters the antagonistic effect of ACh on β-adrenergically stimulated inotropic and lusitropic responses, modifies contraction and relaxation kinetics, and post-rest potentiation of PM contraction. Importantly, this modulatory effect shows pronounced frequency dependence, indicating that ADMA may influence autonomic regulation of myocardial responses under conditions of varying cardiac workload. These findings identify ADMA as a previously unrecognized modulator of ventricular contractility, with particularly prominent effects on autonomic regulation, and suggest a potential mechanism through which elevated ADMA levels may contribute to cardiac dysfunction in cardiovascular disease.

## Methods

### Animals

Adult male Wistar rats (3–4 months old, 300–350 g body weight) were used in this study. Every effort was made to minimize animal suffering, and all operations were performed under isoflurane anesthesia. This study did not involve endangered or protected species and was performed in accordance with Directive 2010/63/EU of the European Parliament. All methods and protocols used in this study were approved by the Animal Care and Use Committee of the University of Wisconsin–Madison and were conducted in accordance with the Guide for the Care and Use of Laboratory Animals published by the National Institutes of Health (NIH; publication no. 85 − 23, revised 1996). All animals received humane care in compliance with these guidelines (protocol no. M005490-R03-A01). Wistar rats (*Rattus norvegicus*) were obtained from Charles River Laboratories (USA).

### Contraction measurements

Papillary muscles (PMs) were excised from the right ventricle of anesthetized rat hearts. Mechanical activity was assessed using a system based on PowerLab 8/35 (ADInstruments) and a force transducer (MLT0420). Isometric force measurements were performed in oxygenated Tyrode solution (95% O_2_ / 5% CO_2_) containing (in mM): NaCl 135, KCl 4, MgCl_2_ 1, CaCl_2_ 1.8, NaHCO_3_ 13.2, NaH_2_PO_4_ 1.8, and glucose 11 (pH 7.4), as previously described (Averin et al. 2022, 2024). To calibrate the system, a load of 1 g was applied. The consistency of the recordings was checked before each experiment.

Briefly, freshly isolated PMs were mounted horizontally in a temperature-controlled chamber (30 ± 0.1°C) and stretched to the length at which maximal contractile force was achieved. Stimuli were applied as rectangular pulses with a duration of 5 ms and with an amplitude 25% above the excitation threshold, for which bipolar Ag-AgCl electrodes were used. Before each experiment, PMs were stimulated at a frequency of 0.3 Hz for 2 hours until complete mechanical stabilization. In ADMA group, the bathing solution contained 10 μM ADMA throughout the stabilization period and all subsequent experimental protocols. Muscles exhibiting autorhythmic activity, mechanical instability, or a contractile force < 1 mN were excluded from the study. The following parameters were recorded: the force of contraction, time to peak tension, time to 50% and 95% relaxation, as well as (dF/dt)_max_ and (dF/dt)_min_, which represent the maximum rates of force development during contraction and force decay during relaxation, respectively. To measure FFR, after complete mechanical stabilization, the stimulation frequency was increased stepwise to 0.1, 0.3, 1.0, and 3.0 Hz. Post-rest potentiation was evaluated at a stimulation frequency of 1 Hz, with rest intervals ranging from 2 to 120 s. After each rest period, the preparation was stimulated until full mechanical recovery of contractile function was achieved.

### Drugs and solutions

All drugs were commercially available and of the highest purity (Sigma Chemical, St. Louis, MO, USA). All compounds were dissolved and diluted in distilled water.

### Data analysis and statistics

All recordings were performed as paired measurements, in which two PMs (control and ADMA-treated) were isolated from the same heart (n = 8–10 animals per experimental parameter) and recorded simultaneously. Two-way repeated measures (RM) ANOVA followed by Sidak’s multiple comparison post-hoc test was used for multiple-group comparisons to evaluate the effects of ISO and ACh in control and ADMA-treated groups during repetitive electrical stimulation at increasing frequencies (0.1, 0.3, 1, and 3 Hz). A p-value < 0.05 was considered statistically significant. All data are presented as mean ± standard error of the mean (SEM).

## Results

### Effects of ADMA on PM contractile force and the force-frequency relationship

First, we investigated whether pretreatment with 10 μM ADMA for 2 h before and throughout the experiment affected the baseline force-frequency relationship (FFR) of PM contractility (Fig. 1a). Continuous exposure to ADMA did not affect the FFR, as the main effect of ADMA treatment was not significant with the two-way RM ANOVA (F(1,9) = 0.74, P > 0.05; Fig. 1). However, a subsequent analysis with Sidak’s post-hoc test revealed that treatment with ADMA significantly decreased PM force of contraction at the 3 Hz stimulation frequency (CI_95%_ [0.27; 1.39], P < 0.01; Fig. 1B). These results indicate that ADMA at a concentration of 10 μM does not affect basal FFR but rather exerts a negative inotropic effect at high stimulation frequency.

Next, we investigated how 1 μM isoproterenol (ISO) and 10 μM acetylcholine (ACh) affected contractile force and the FFR of untreated PMs (control) and PMs pretreated with 10 μM ADMA. We found that both ISO and ACh produced comparable effects on contractility in both control and ADMA groups (F(2,18) = 11.97, P < 0.0005 with two-way RM ANOVA for treatment in control group and F(2,18) = 10.42, P < 0.001 with two-way RM ANOVA for treatment in ADMA group; Fig. 2e and f), but there was a significant difference in the efficacy of ACh antagonism against ISO in the ADMA group (Fig. 2a and b). Thus, in both groups, ISO produced a positive inotropic effect at 0.1–1 Hz frequency range, and at the 3 Hz stimulation frequency the force of PM contraction under ISO was similar to the baseline levels due to the positive inotropic effect of 3 Hz stimulation under baseline conditions (F(6,54) = 6.75, P < 0.0005 with two-way RM ANOVA for frequency and treatment interaction in the control group and F(6,54) = 8.33, P < 0.0001 with two-way RM ANOVA for frequency and treatment interaction in the ADMA group; Fig. 2e and f).

ACh significantly reversed the ISO-induced positive inotropic effect returning PM contractile force close to basal levels in the control group for 0.1 Hz (CI_95%_ [0.29; 1.1], P < 0.0001 for ACh versus ISO and CI_95%_ [−0.54; 0.28], P > 0.05 for ACh versus baseline with Sidak’s post-hoc test; Fig. 2E, F), 0.3 Hz (CI_95%_ [0.38; 1.2], P < 0.0001 for ACh versus ISO and CI_95%_ [−0.72; 0.08], P > 0.05 for ACh versus baseline with Sidak’s post-hoc test; Fig. 2e and f), and 1 Hz (CI_95%_ [0.17; 0.99], P < 0.001 for ACh versus ISO and CI_95%_ [−0.53; 0.28], P > 0.05 for ACh versus baseline with Sidak’s post-hoc test; Fig. 2e and f) stimulation frequencies and did not exert any significant effect at 3 Hz as compared to baseline and ISO (P > 0.05 for both ACh versus ISO and ACh versus baseline with Sidak’s post-hoc test; Fig. 2E, F)e and f).

Incubation with ADMA significantly weakened the inhibitory effect of ACh on ISO-induced positive inotropy at 0.3 Hz as PM contractile force in the presence of ACh remained significantly higher than baseline (CI_95%_ [0.34; 1.1], P < 0.0001 for ACh versus ISO and CI_95%_ [−0.76; −0.01], P < 0.05 for ACh versus baseline with Sidak’s post-hoc test; Fig. 2E, Fe and f) while at the high stimulation frequency of 3 Hz ACh produced a negative inotropic effect significantly reducing force of PM contraction compared with both baseline and ISO (CI_95%_ [0.08; 0.83], P < 0.01 for ACh versus ISO and CI_95%_ [0.08; 0.83], P < 0.01 for ACh versus baseline with Sidak’s post-hoc test; Fig. 2e and f).

### Effects of ADMA on the maximal rates of PM contraction and relaxation

As treatment with ADMA affected the ability of ACh to reverse the positive inotropic effect of ISO on PM contractile force and the FFR, we next investigated whether ADMA also affects the maximal rates of PM contraction (+ dF/dtmax) and relaxation (-dF/dtmax). Similar to contractile force, ISO increased + dF/dtmax of PM in control and ADMA groups over 0.1–1 Hz frequency range and had no effect at 3 Hz due to the increased baseline inotropic response to 3 Hz stimulation (F(6,46) = 5.1, P < 0.001 with two-way RM ANOVA for frequency and treatment interaction in control group and F(6,54) = 7.4, P < 0.0001 with two-way RM ANOVA for frequency and treatment interaction in the ADMA group; Fig. 3a and c). ISO also potentiated -dF/dt_max_ of PM relaxation in control and ADMA-treated groups (F(6,46) = 5.7, P < 0.001 with two-way RM ANOVA for frequency and treatment interaction in the control group and F(6,54) = 5.8, P < 0.0001 with two-way RM ANOVA for frequency and treatment interaction in the ADMA group; Fig. 3a and c). Interestingly, for -dF/dt_max_, the effect of ISO at 3 Hz stimulation frequency was still significantly higher compared to baseline in ADMA-treated group (CI_95%_ [−1.26; −0.08], P < 0.05 for baseline versus ISO with Sidak’s post-hoc test; Fig. 3d) and, similarly to + dF/dt_max_, there was no difference from baseline at 3 Hz in the control group for -dF/dt_max_ (P > 0.05 with Sidak’s post-hoc test; Fig. 3b for ISO versus baseline). In the control group, ACh was able to reverse ISO action returning + dF/dt_max_ and -dF/dt_max_ to baseline levels for all tested stimulation frequencies (P > 0.05 with Sidak’s post-hoc test for ACh versus baseline; Fig. 3a and c). Incubation with ADMA weakened the antagonistic action of ACh against ISO at 0.3 Hz, as at this stimulation frequency ACh was not able to bring ISO potentiated + dF/dt to the baseline level (CI_95%_ [−0.87; −0.09], P < 0.005 for baseline versus ACh with Sidak’s post-hoc test; Fig. 3c). At 3 Hz, ADMA potentiated the negative effect of ACh on + dF/dt_max_ of PM contraction as ACh was able to significantly decrease + dF/dt_max_ compared to both baseline and ISO levels (CI_95%_ [0.014; 0.78], P < 0.05 for baseline versus ACh and CI_95%_ [0.16; 0.93], P < 0.01 for ISO versus ACh with Sidak’s post-hoc test; Fig. 3c). For maximal rate of relaxation, ADMA weakened the ability of ACh to reverse the ISO-induced increase in -dF/dt_max_ of PM at 0.1 Hz (CI_95%_ [−1.24; −0.3], P < 0.05 for baseline versus ACh with Sidak’s post-hoc test; Fig. 3d) and 0.3 Hz (CI_95%_ [−1.48; −0.06], P < 0.0005 for baseline versus ACh with Sidak’s post-hoc test; Fig. 3d) stimulation frequencies and enhanced the antagonistic effect of ACh against ISO at 3 Hz (CI_95%_ [0.18; 1.36], P < 0.05 for ISO versus ACh with Sidak’s post-hoc test; Fig. 3d).

#### The effects of ADMA on kinetics of PM contraction and relaxation.

Next, we investigated how ADMA affected ISO and ACh effects on the kinetics of PM contraction and relaxation by measuring time to peak tension (TPT) and time from peak to 50% and 95% relaxation (RT50% and RT95%, respectively). We found that ISO exerted a negative lusitropic effect, significantly decreasing TPT (F(2,14) = 45.6, P < 0.0001 with two-way RM ANOVA for treatment; Fig. 4a), RT50% (F(2,14) = 147.6, P < 0.0001 with two-way RM ANOVA for treatment; Fig. 4b), and RT95% (F(2,14) = 281.2, P < 0.0001 with two-way RM ANOVA for treatment; Fig. 4c) in the control group across all tested stimulation frequencies. Similar to control, ISO exerted a negative lusitropic effect on TPT (F(2,16) = 42.6, P < 0.0001 with two-way RM ANOVA for treatment; Fig. 4d), RT50% (F(2,14) = 77.8, P < 0.0001 with two-way RM ANOVA for treatment; Fig. 4e), and RT95% (F(2,12) = 256.5, P < 0.0001 with two-way RM ANOVA for treatment; Fig. 4f) in the ADMA-treated group. In the control group, ACh did not antagonize the negative lusitropic effect of ISO as the values for TPT, RT50%, and RT95% under ACh did not differ from those under ISO across all tested stimulation frequencies (P > 0.05 with Sidak’s post-hoc test for ISO versus ACh; Fig. 4a, b, c).

In the ADMA-treated group, similarly to the control group, negative lusitropic action of ISO on TPT and RT50% was not antagonized by ACh (P > 0.05 with Sidak’s post-hoc test for ISO versus ACh; Fig. 4d, e, f). However, in the ADMA-treated group, ACh slightly but significantly weakened the negative lusitropic effect of ISO on PM contractility for RT95% at 0.3 Hz (CI_95%_ [−0.02; −0.003], P < 0.01 for ISO versus ACh with Sidak’s post-hoc test; Fig. 4f), 1 Hz (CI_95%_ [−0.02; −0.001], P < 0.05 for ISO versus ACh with Sidak’s post-hoc test; Fig. 4f), and 3 Hz (CI_95%_ [−0.02; −0.0001], P < 0.05 for ISO versus ACh with Sidak’s post-hoc test; Fig. 4f) stimulation frequencies.

#### Effects of ADMA on the post-rest potentiation of PM contractility.

Finally, we assessed how ADMA affected the post-rest potentiation (PRP) of PM contractility at a variety of inter-stimulation rest intervals. We found that baseline PRP increased with increasing inter-stimulation intervals. Compared to baseline, ISO significantly increased the PRP at short rest intervals of 2 and 5 seconds and decreased it at intervals of 30 seconds and longer, while ACh significantly decreased the PRP at intervals of 10 seconds and longer (Fig. 5a and b). This pattern of ISO and ACh effects was similar in control and ADMA-treated PM preparations (F(14,126) = 15.17, P < 0.0001 with two-way RM ANOVA for frequency and treatment interaction in the control group and F(14,126) = 13.62, P < 0.0001 with two-way RM ANOVA for frequency and treatment interaction in the ADMA group; Fig. 5c and d). Subsequent analysis revealed that ADMA treatment attenuated the potentiating effect of ISO on the PRP at the 5-second rest interval. Thus, in the control group, ISO significantly increased the PRP compared to baseline (CI_95%_ [−0.732; −0.061], P < 0.01 for ISO versus baseline with Sidak’s post-hoc test; Fig. 5c) and ACh (CI_95%_ [0.1927; 0.8641], P < 0.001 for ISO versus ACh with Sidak’s post-hoc test; Fig. 5c) values.

In the ADMA-treated group, ISO did not affect the PRP compared to baseline (P > 0.05 with Sidak’s post-hoc test for ISO versus baseline; Fig. 5D) while still showing its potentiating effect compared to ACh (CI_95%_ [0.1927; 0.8641], P < 0.001 for ISO versus ACh with Sidak’s post-hoc test; Fig. 5d). Analysis of the PRP at the 15-second inter-stimulation interval showed that ADMA enhanced the negative effect of ACh on the PRP. Thus, in the control group, ACh significantly decreased the PRP compared to baseline (CI_95%_ [0.242; 0.913], P < 0.001 for ACh versus baseline with Sidak’s post-hoc test; Fig. 4d) and ACh did not have any effect on the PRP compared to ISO (P > 0.05 with Sidak’s post-hoc test for ISO versus ACh; Fig. 4c). In the ADMA-treated group, ACh significantly decreased the PRP compared to both baseline (CI_95%_ [0.375; 1.036], P < 0.0001 for ISO versus ACh with Sidak’s post-hoc test; Fig. 4d) and ISO (CI_95%_ [0.125; 0.786], P < 0.001 for ISO versus ACh with Sidak’s post-hoc test; Fig. 4d) values.

## Discussion

In the present study, we investigated how ADMA affects the autonomic regulation of cardiac contractility. First, we found that treatment with ADMA attenuated basal PM contractile force at high stimulation frequency of 3 Hz, without affecting the overall FFR. Next, we showed that continuous incubation with ADMA significantly affected the ability of ACh to antagonize ISO-driven stimulation of rat right ventricular PM contractility. We found that ADMA can modulate the antagonistic effect of ACh on ISO-driven positive inotropic and negative lusitropic effects as well as the ISO-induced increase in the maximal rates of contraction and relaxation. The modulatory effect of ADMA showed a frequency-dependent bidirectional nature as ADMA weakened anti-ISO ACh action at low stimulation frequency range and potentiated the antagonistic effect of ACh at high stimulation frequency. In addition, ADMA weakened the potentiating effect of ISO and strengthened the inhibitory effect of ACh on the post-rest potentiation of PM contractility.

Xiong and colleagues reported that exogenous ADMA reduces rat left ventricle PM contractility in a dose- and time-dependent manner (Xiong et al. 2021). It was found that incubation of PM with 10 μM ADMA did not have a significant effect on contraction force of PM stimulated at 1 Hz frequency while an increase in ADMA concentration up to 30 μM and higher exerted a profound negative inotropic effect (Xiong et al. 2021). In our work, consistent with the findings of Xiong et al., 10 μM ADMA did not affect PM force of contraction at 1 Hz stimulation frequency but there was a significant reduction in PM contractility in the ADMA-treated group compared to untreated control, when stimulation frequency was increased up to 3 Hz. Several studies indicated that the activity of nitric oxide synthase (NOS) is critical for the frequency-dependent potentiation of cardiac contractility and that in rodents the contribution of NOS to the positive FFR increased with elevation of stimulation frequency (Kaye et al. 1996; Khan et al. 2003; Langer et al. 2003; Dedkova et al. 2007). These findings allowed us to suggest that ADMA, when added at a concentration similar to that observed at intracellular levels (Teerlink et al. 2009), could exert a negative inotropic effect on PM contractility at high stimulation frequency due to its inhibitory action on NOS, which is itself critical for the maintenance of the PM positive FFR at high frequency stimulation rates. Next, we showed that the effect of 10 μM ADMA can be further revealed when PMs were examined during autonomic regulation by β-adrenergic and muscarinic stimulation. We found that, in pharmacologically stimulated PMs, ADMA exerted a modulatory effect on anti-β-adrenergic effects of ACh. In this case, depending on the frequency of PM stimulation, ADMA either weakened or potentiated the anti-adrenergic effect of ACh. We suggest that in this case ADMA may interfere with β-adrenergic-driven stimulation of NOS activity in cardiomyocytes. Previous studies have shown that β-adrenergic stimulation plays a critical role in the regulation of cardiac NOS activity; however, this area of research remains complex and incompletely understood. Several studies have shown that exogenous NO donors do not affect cardiac contractility, both under baseline conditions and β-adrenergic stimulation (Weyrich et al. 1994; Wyeth et al. 1996; Worthley et al. 2005). However, other studies revealed that NO donors can exert positive inotropic effects on cardiac contractility under basal conditions as well as during β-adrenergic stimulation and that absent/weak effect of NO donors in some studies might be related to the fact that cardiac contractility is already maintained by near-saturating endogenous NO levels (Finkel et al. 1995; Prendergast et al. 1998; Müller-Strahl et al. 2000; Reading and Barclay 2002; Vielma et al. 2016). In support to this, several studies utilizing knock-outs of two types of NOS known for their expression in the cardiomyocytes – NOS1 (nNOS) and NOS3 (eNOS), showed that NOS plays a critical role in the maintenance of cardiac contractility under baseline conditions and during β-adrenergic stimulation. Most of these studies with knock-out animals indicate that neither NOS1 nor NOS3 are involved in the anti-β-adrenergic effects of muscarinic stimulation (Vandecasteele et al. 1999; Gödecke et al. 2001; Brunner et al. 2001; Ashley et al. 2002; Sears et al. 2003; Martin et al. 2006). So far, there is only one study that, by using eNOS^null^ (NOS3^null^) mice, showed that eNOS plays a critical role in muscarinic receptor-mediated negative inotropic effect against ISO-induced potentiation of cardiomyocyte shortening in response to electrical stimulation (Han et al. 1998). This study also showed that muscarinic effects on ISO-potentiated cardiomyocyte shortening depended on an NO-mediated decrease in the L-type Ca^2+^ current (*I*_Ca_) (Han et al. 1998). However, other studies indicate that a negative inotropic effect of muscarinic receptor activation is mediated via G_i_ protein-mediated reduction in adenylate cyclase activity rather than NO signaling. It has been found that the NOS inhibitor L-NAME can reduce carbachol-driven stimulation of NO, while it has no effect on negative inotropic effects of carbachol on forskolin-stimulated PM contractility (Hui et al. 1995). Furthermore, it is an inhibition of G_i_ protein with pertussis toxin, rather than inhibition of the NO-cGMP pathway, that mediates the negative inotropic effect of muscarinic agonist carbachol in rat cardiomyocytes (Sandirasegarane and Diamond 2003). Other studies indicate that it is the interplay between NO signaling and the β-adrenergic system that can affect cardiac contractility. It was found that NO-cGMP signaling activators can exert a synergistic positive inotropic effect on PM contractility when co-administered with low doses of β_1_-adrenergic receptor agonist dobutamine (Reading and Barclay 2002). Meanwhile, when PM contractility was stimulated with high doses of dobutamine, activators of NO-cGMP signaling antagonized the positive inotropic effect of this β_1_-adrenergic receptor agonist (Reading and Barclay 2002). Another example of modulatory action of NO signaling was found in a study where activators of NO signaling were acting synergistically with ACh by inhibiting ISO-potentiated *I*_Ca_ in rat ventricular cardiomyocytes. In that study, activators of NO signaling alone did not affect ISO-induced potentiation of *I*_Ca_ and ACh was able to only partially inhibit ISO-potentiated *I*_Ca_ while *I*_Ca_ returned to basal levels when activators of NO signaling and ACh were applied together (Grushin et al. 2008).

Considering these facts and that the baseline level of PM contractility depends on NO production and NOS activity in a frequency-dependent manner (Kaye et al. 1996; Khan et al. 2003; Langer et al. 2003; Dedkova et al. 2007), we suggest that differences in the effects of ADMA on the antagonistic effect of ACh on ISO-induced modulation of PM contractility at low and high stimulation frequencies could be attributed to the difference in the basal NO levels. We further suggest that a low stimulation frequency can cause moderate NO production that can act synergistically with ACh, for instance, reducing a positive inotropic effect of ISO via modulation of *I*_Ca_, and ADMA might potentially weaken this synergistic effect by inhibiting NOS (Balligand et al. 1995; Belhassen et al. 1996; Grushin et al. 2008). In turn, a high stimulation frequency may be associated with excessive NO production that can lead to NO-mediated positive inotropic effects at baseline and act synergistically with ISO by causing a negative lusitropic effect. In this case, ADMA could potentiate the antagonistic effect of ACh towards ISO by inhibiting high stimulation-driven excessive NO production at baseline (Ashley et al. 2002; Khan et al. 2003). However, this hypothesis requires further investigation. It would be critical to study the efficacy of ADMA-mediated inhibition of both NOS1 and NOS3 isoforms, as they play partially distinct roles in the regulation of cardiac calcium handling and contractility and show cardiomyocyte-specific compartmentalization, with NOS1 being expressed in sarcoplasmic reticulum and NOS3 localized on the cardiomyocyte sarcolemma (Dedkova et al. 2007; Ziolo et al. 2008). In the present study, we showed that ADMA weakened ISO-driven increase in the PRP and further strengthened the reduction in the PRP mediated by ACh. PRP reflects the ability of the sarcoplasmic reticulum to reuptake and release Ca^2+^ (Koch-Weser and Blinks 1963; Allen et al. 1976; Bers 1987; Pieske et al. 1996), suggesting that ADMA may influence NOS1-mediated regulation of SR Ca^2+^ load and release, thus affecting PM force of contraction. However, this hypothesis requires detailed investigation.

In conclusion, our study demonstrates that ADMA is significantly involved in autonomic regulation of cardiac contractility by β-adrenergic and muscarinic systems while showing moderate negative inotropic effect on basal PM contractility only at high stimulation frequency. A large number of studies showed that elevated ADMA concentrations are associated with the development of heart failure, cardiac hypertrophy, and cardiomyopathy (Wilson Tang et al. 2008; Nemeth et al. 2015; Cordts et al. 2019). On the other hand, there is notable exception, namely Takotsubo cardiomyopathy, which is associated with NO overproduction due to low ADMA levels (Nguyen et al. 2013). Animal models of Takotsubo cardiomyopathy show a decreased myocardial contractile function, a negative effect of β-adrenergic stimulation on cardiac outcome in stressed animals, and the involvement of muscarinic signaling in the regulation of stress tolerance (Kurbatov et al. 2021). Collectively, these studies indicate that the maintenance of physiological ADMA levels is critical for cardiac function as imbalances in ADMA levels can lead to either NO deficiency or NO overproduction. Our results further expand previous findings showing that ADMA could exert bidirectional action on myocardial contractility as it can either potentiate the anti-β-adrenergic effect of muscarinic mediator ACh on PM contractility at high stimulation frequencies or weaken ACh effects on ISO-driven potentiation of PM contraction at low-to-moderate stimulation frequencies. Our results provide new evidence for endothelial-independent mechanism by which ADMA contributes to the regulation of cardiac function.

## Figures and Tables

**Figure 1:**
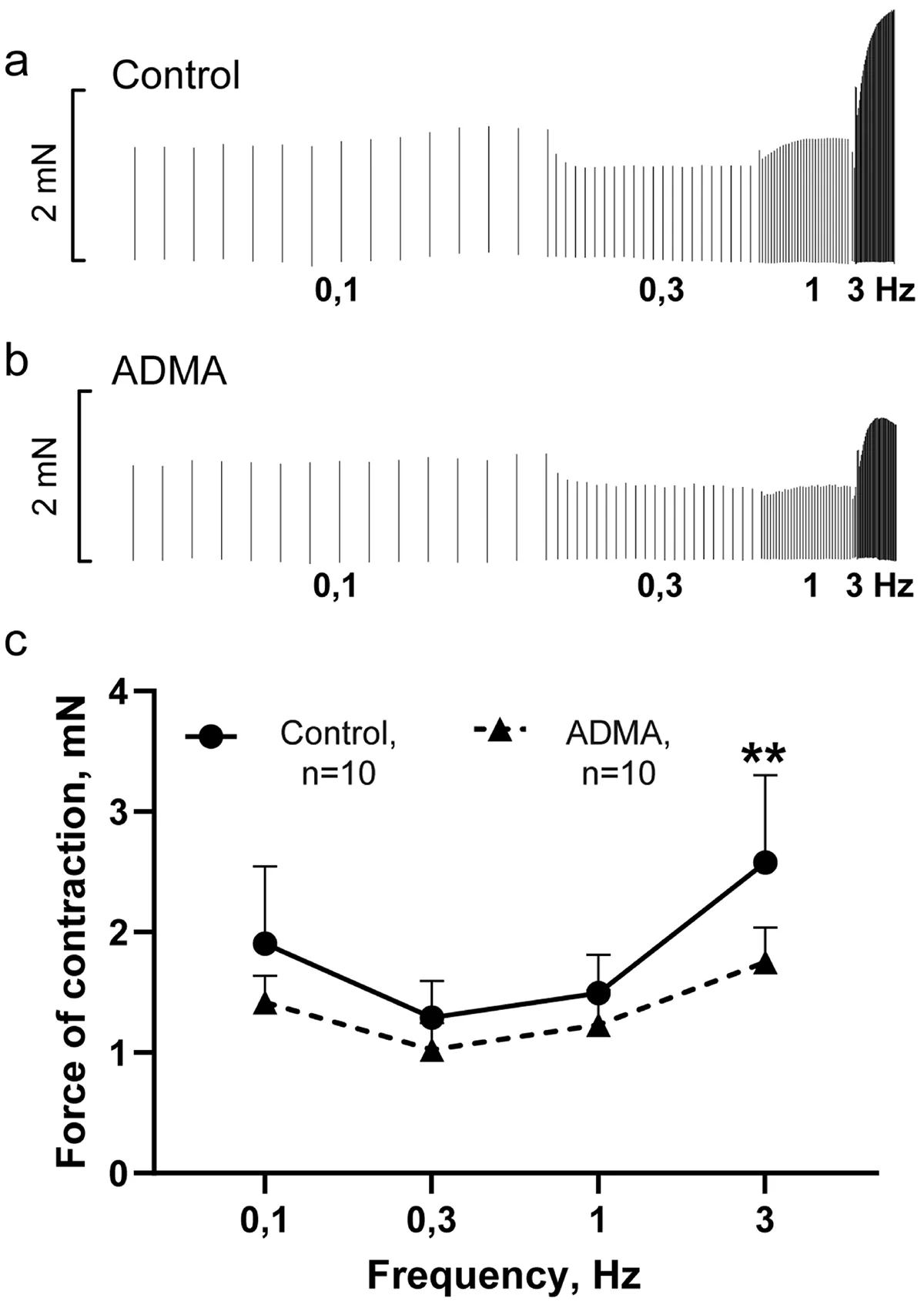
ADMA effects on PM baseline contractility and the force-frequency relationship (FFR). Panels **a** and **b**show representative traces of PM force of contraction at 0.1, 0.3, 1, and 3 Hz stimulation frequencies in control (**a**) and ADMA-treated (**b**) groups. Panel **c** shows the FFR for control (*circles*) and ADMA-treated (*triangles*) groups. The data is presented as means ± SEM. **p<0.01 with Sidak’s post-hoc test.

**Figure 2:**
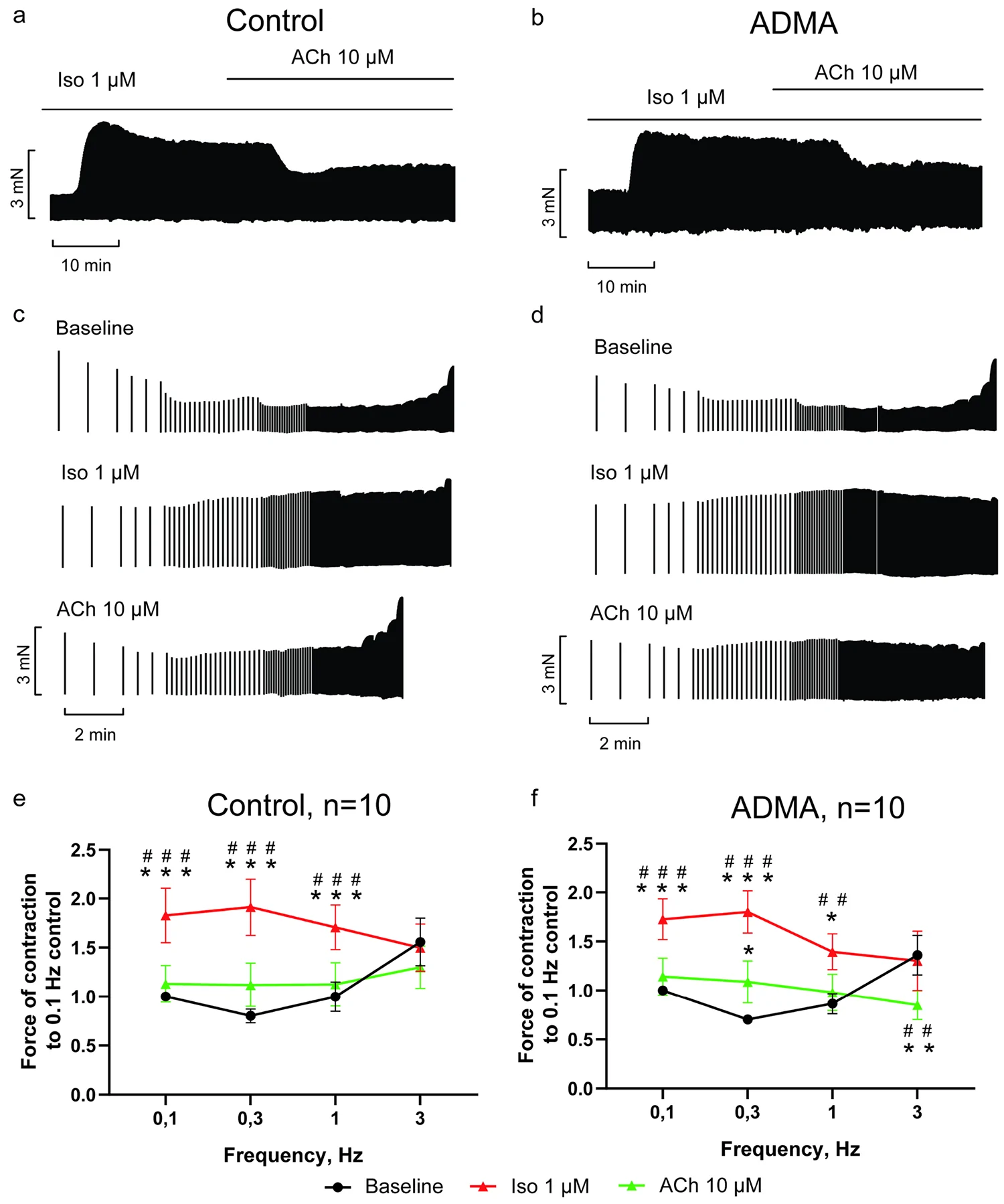
Effects of ISO and ACh on PM force of contraction and the force-frequency relationship (FFR) in control and ADMA-treated groups. Panels **a** and **b** show representative traces of PM force of contraction at 0.3 Hz stimulation frequency in control (**a**) and ADMA (**b**) groups. Panels **c** and **d** show representative traces of the FFR for control (**c**) and ADMA (**d**) groups. Panels **e** and **f** show FFR curves for control (**e**) and ADMA (**f**) groups. The data is presented as means ± SEM. * shows comparison between baseline and ISO or ACh while # compares ISO versus ACh. *p < 0.05; **p < 0.01; *** p < 0.0001 and ^#^p < 0.05; ^###^p < 0.01 and ^###^p < 0.0001 with two-way RM ANOVA followed by Sidak’s post-hoc test.

**Figure 3:**
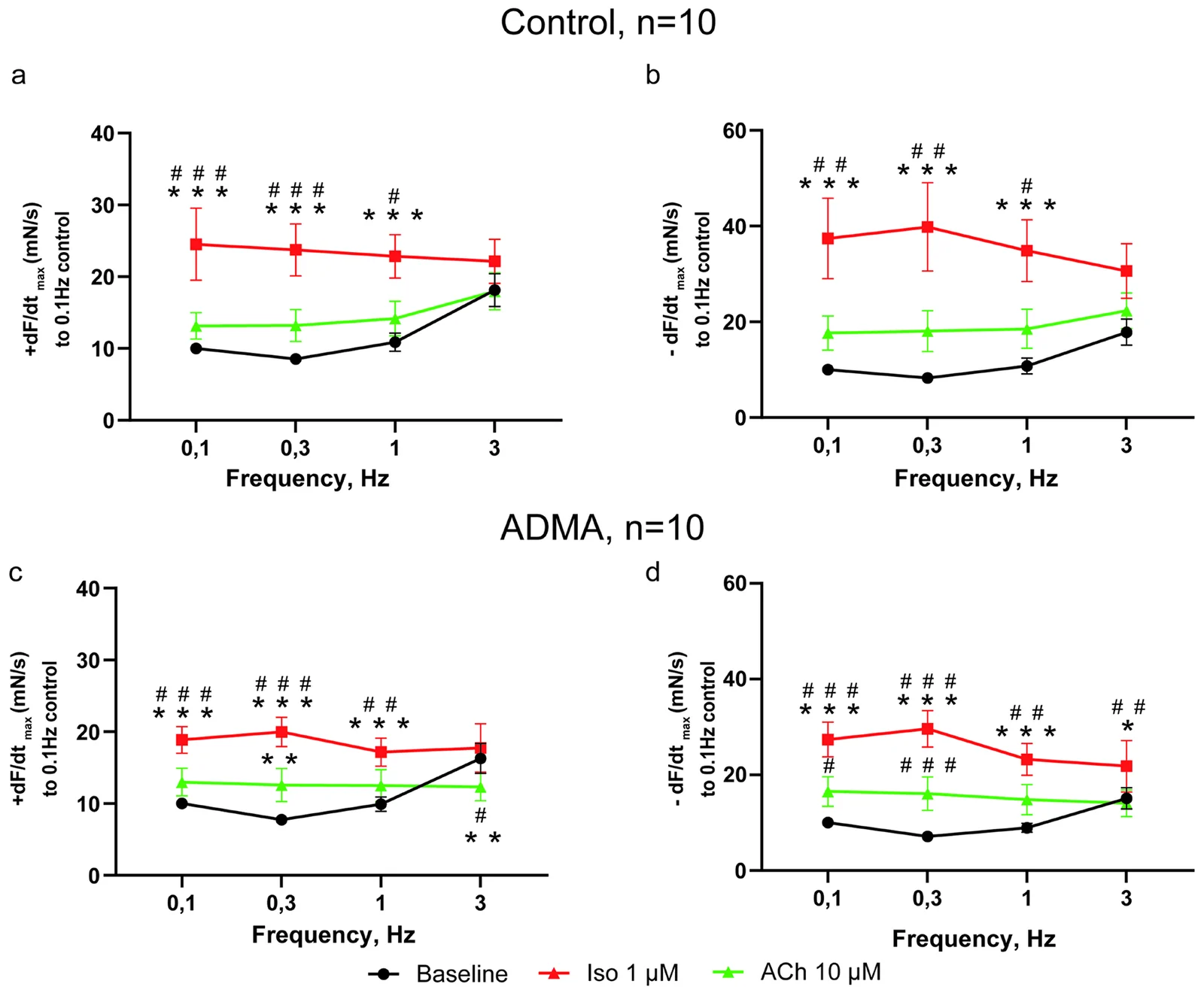
Effects of ISO and ACh on maximum force of contraction, (+dF/dt)_max_, and relaxation, (-dF/dt)_max_, in control and ADMA-treated groups. Panels **a** and **b** show +dF/dt_max_ (**a**) and -dF/dt_max_ (**b**) for control, and panels **c** and **d** show +dF/dt_max_ (**c**) and -dF/dt_max_ (**d**) for ADMA-treated groups. The data is presented as means ± SEM. * shows comparison between baseline and ISO or ACh while # compares ISO versus ACh. *p < 0.05; **p < 0.01; *** p < 0.0001 and ^#^p < 0.05; ^###^p < 0.01 and ^###^p < 0.0001 with two-way RM ANOVA followed by Sidak’s post-hoc test.

**Figure 4:**
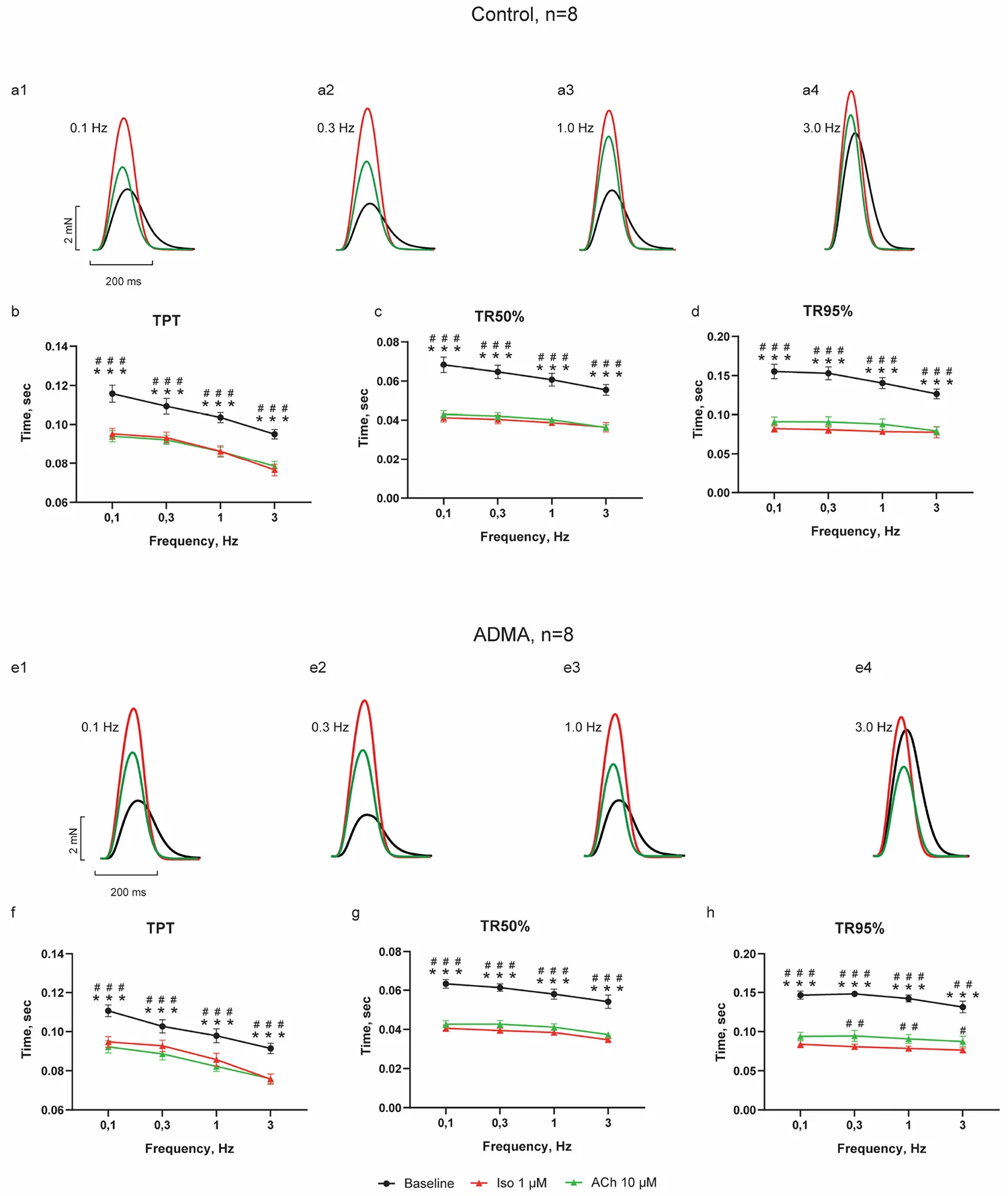
Effects of ISO and ACh on the kinetic parameters of contraction in PMs in control and ADMA-treated groups. Panels **a1-a4** and **e1-e4** show representative traces for kinetics of PM contraction for the baseline and under ISO and ACh administration taken at 0.1 (**a1 and e1**), 0.3 (**a2 and e2**), 1 (**a3 and e3**), and 3 Hz (**a4 and e4**) stimulation frequencies for control (**a1-a4**) and ADMA (**e1-e4**) groups. Panels **b** and **f** show results for time to peak tension (TPT) of the maximal contraction in control (**b**) and ADMA-treated (**f**) groups. Panels **c** and **g** show results for time from peak to 50% of the maximal contraction (TR50%) in control (**c**) and ADMA-treated (**g**) groups. Panels **d** and **h** show results for time from peak to 95% of the maximal contraction (TR95%) in control (**d**) and ADMA-treated (**h**) groups. The data is presented as means ± SEM. * shows comparison between baseline and ISO or ACh, while # compares ISO versus ACh. *** p < 0.0001 and ^#^p < 0.05; ^###^p < 0.01 and ^###^p < 0.0001 with two-way RM ANOVA followed by Sidak’s post-hoc test.

**Figure 5:**
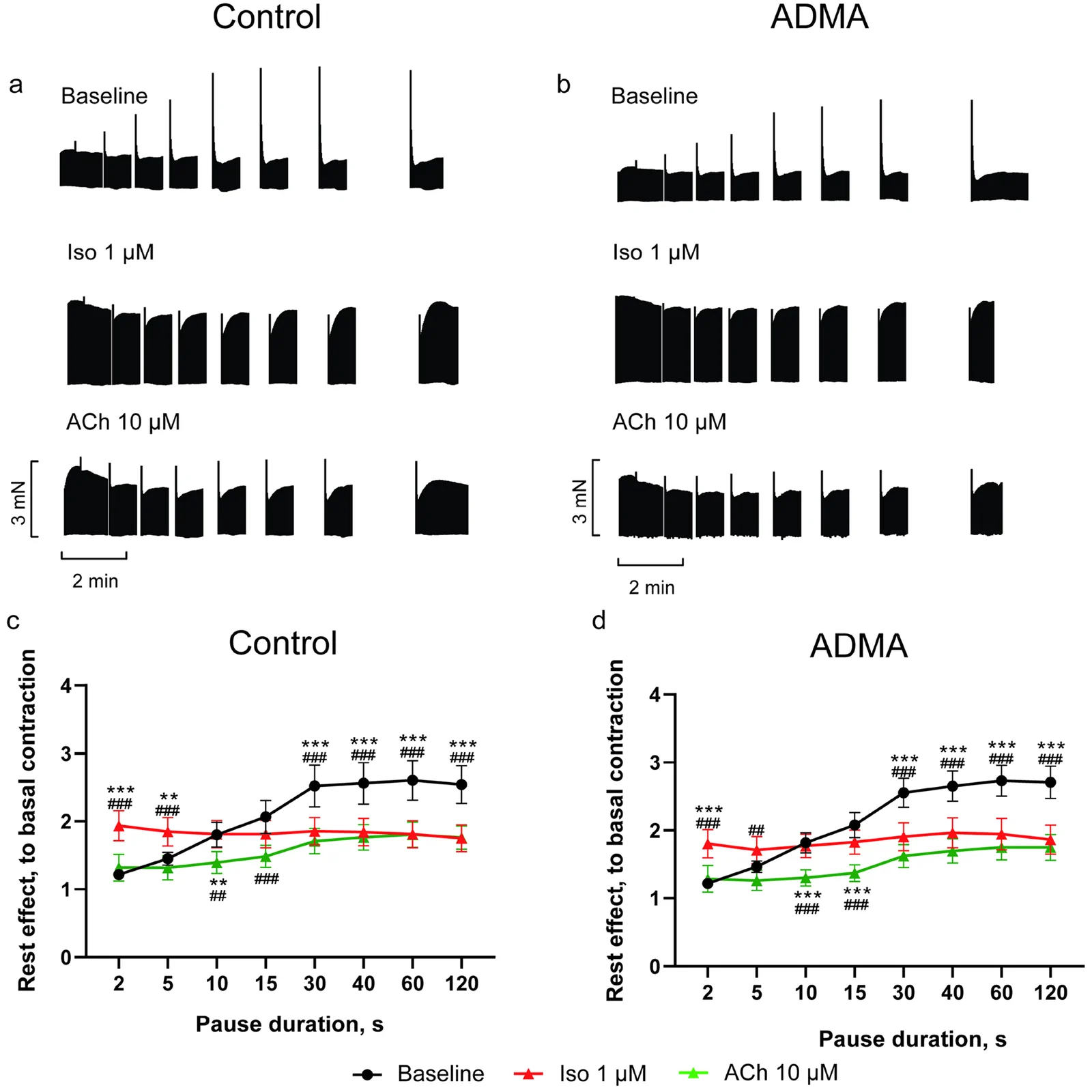
Effects of ISO and ACh on the post-rest potentiation (PRP) in control and ADMA-treated groups. Panels **a** and **b** show representative recordings of the PRP at different time intervals at baseline and under ISO and ACh administration in control (**a**) and ADMA-treated groups (**b**). Panels **c** and **d** show results for the rest effect on PM contractility in control (**c**) and ADMA-treated (**d**) groups. The data is presented as means ± SEM. * shows comparison between baseline and ISO or ACh, while # compares ISO versus ACh. **p < 0.01; *** p < 0.0001 and ^###^p < 0.01 and ^###^p < 0.0001 with two-way RM ANOVA followed by Sidak’s post-hoc test.

## Data Availability

Individual values of all supporting data are available upon request.
